# Supplementary material for: Covid-19 vaccine effectiveness against general SARS-CoV-2 infection from the omicron variant: A retrospective cohort study
Source: PLOS Glob Public Health. 2023 Jan 10;3(1):e0001111. doi: 10.1371/journal.pgph.0001111 (PMC9910751; doi:10.1371/journal.pgph.0001111)
Supplement: S1 Appendix — (DOCX) [file pgph.0001111.s001.docx]

# **S1 Appendix. Statistical models for vaccine protection (Table 2)**

**Vaccine protection (across manufacturers):**

$h\left( t | V_{i},P_{i},\boldsymbol{X}_{i} \right)=h_{0}\left( t \right)\times\exp\left\{ \alpha\times V_{Pi}\left( t \right)+\beta\times V_{Fi}\left( t \right)+\gamma\times V_{Bi}(t)+\nu\times P_{i}+\boldsymbol{\eta}'\boldsymbol{X}_{i} \right\}$ (Model 1.1) ^†^

- Protection from full vaccination: $1-\exp\left\{ \beta\right\}$
- Protection from booster dose: $1-\exp\left\{ \gamma\right\}$
  - Increase in protection from booster dose: $\exp\left\{ \beta\right\}-\exp\left\{ \gamma\right\}$
- $V_{Pi}(t)$ = 1 if subject *i* is partially vaccinated at time *t*, and 0 otherwise (partially vaccinated = 14 days past first dose of mRNA-1273 or BNT162b2 and without second dose by time *t*)
- $V_{Fi}(t)$ = 1 if subject *i* is fully vaccinated at time *t*, and 0 otherwise (fully vaccinated = 14 days past second dose of mRNA-1273 or BNT162b2)
- $V_{Bi\left( t \right)}=1$ if subject *i* is boosted at time t, and 0 otherwise (booster = 7 days past booster dose of mRNA-1273 or BNT162b2)
- $P_{i}$=1 if subject *i* has previous SARS-CoV-2 infection occurring prior to the follow-up period, and 0 otherwise.
  - Note that $P_{i}$ remains time-invariant in this setting since it is not possible to have a previous infection occur during the follow-up period (since this is the event of interest and would therefore be classified as an infection during follow-up).

For employees, $\boldsymbol{X}_{i}$ = covariate vector for subject *i*, and includes age, race/ethnicity (categories defined in Table 1), gender (categories defined in Table 1), affiliation status (faculty/staff), self-reported presence of any of the following conditions: high blood pressure, heart disease, diabetes, overweight or obesity, kidney disease or dialysis, previous stroke or other neurological condition affecting my ability to cough, liver disease, or lung disease, self-reported use of tobacco or nicotine products, and number of SARS-CoV-2 tests since Fall 2020. For students, $\boldsymbol{X}_{i}$ also includes indicator for graduate student status subject, and affiliation status is (residential housing/non-residential housing).

**Vaccine protection (by manufacturer)**

$h\left( t | \boldsymbol{V}_{i},P_{i},\boldsymbol{X}_{i} \right)=h_{0}\left( t \right)\times\exp\left\{ \begin{aligned} \sum_{k=1:2} \alpha_{k}\times V_{P_{k},i}\left( t \right)+\sum_{l=1:2} \beta_{l}\times V_{F_{l},i}\left( t \right)+\sum_{j=1:2} \gamma_{j}\times V_{B_{j},i}\left( t \right) \\ +\nu\times P_{i}+\boldsymbol{\eta}'\boldsymbol{X}_{i} \end{aligned} \right\}$ (Model 1.2)

- Protection from full vaccination by mRNA-1273: $1-\exp\left\{ \beta_{1} \right\}$
- Protection from full vaccination by BNT162b2: $1-\exp\left\{ \beta_{2} \right\}$
  - Differences in vaccine effectiveness (2-dose series) between mRNA-1273 and BNT162b2 is evaluated by testing the null hypothesis $H_{0}: \beta_{1}=\beta_{2}$
- Protection from booster dose by mRNA-1273: $1-\exp\left\{ \gamma_{1} \right\}$
- Protection from booster dose by BNT162b2: $1-\exp\left\{ \gamma_{2} \right\}$
  - Differences in booster effectiveness between mRNA-1273 and BNT162b2 is evaluated by testing the null hypothesis $H_{0}: \gamma_{1}=\gamma_{2}$.
- $V_{P_{1},i}(t)$ = 1 if subject *i* is partially vaccinated from mRNA-1273 at time *t*, and 0 otherwise
- $V_{P_{2},i}(t)$ = 1 if subject *i* is partially vaccinated from BNT162b2 at time *t*, and 0 otherwise
- $V_{F_{1},i}(t)$ = 1 if subject *i* is fully vaccinated from mRNA-1273 at time *t*, and 0 otherwise
- $V_{F_{2},i}(t)$ = 1 if subject *i* is fully vaccinated from BNT162b2 at time *t*, and 0 otherwise
- $V_{B_{1},i}\left( t \right)$ = 1 if subject *i* is boosted from mRNA-1273 at time t, and 0 otherwise
- $V_{B_{2},i}\left( t \right)$ = 1 if subject *i* is boosted from BNT162b2 at time t, and 0 otherwise

^†^ When estimating the unadjusted protection in Table 2, we set $\boldsymbol{\eta}=\boldsymbol{0}$.

**Vaccine protection for mRNA-1273/BNT162b2 sequence versus mix matched 2-dose/booster**

$h\left( t | \boldsymbol{V}_{i},P_{i},\boldsymbol{X}_{i} \right)=h_{0}\left( t \right)\times\exp\left\{ \begin{aligned} \sum_{k=1:2} \alpha_{k}\times V_{P_{k},i}\left( t \right)+\sum_{l=1:2} \beta_{l}\times V_{F_{l},i}\left( t \right)+\sum_{j=1:2} \gamma_{j}\times V_{B_{1j},i}\left( t \right) \\ \delta\times V_{B_{2},i}(t)+\nu\times P_{i}+\boldsymbol{\eta}'\boldsymbol{X}_{i} \end{aligned} \right\}$ (Model 1.3)

- Protection from complete mRNA-1273 sequence (i.e. 2-dose mRNA-1273 followed by mRNA-1273 booster): $1-exp\{\gamma_{1}\}$
- Protection from complete BNT162b2 sequence (i.e. 2-dose BNT162b2 followed by BNT162b2 booster): $1-exp\{\gamma_{2}\}$
- Protection from mix-matched booster: $1-exp\{\delta\}$
- Difference between mix-matched booster and complete mRNA-1273 sequence: $H_{0}:\delta=\gamma_{1}$
- Difference between mix-matched booster and complete BNT162b2 sequence: $H_{0}:\delta=\gamma_{2}$
- $V_{P_{1},i}(t)$ = 1 if subject *i* is partially vaccinated from mRNA-1273 at time *t*, and 0 otherwise
- $V_{P_{2},i}(t)$ = 1 if subject *i* is partially vaccinated from BNT162b2 at time *t*, and 0 otherwise
- $V_{F_{1},i}(t)$ = 1 if subject *i* is fully vaccinated from mRNA-1273 at time *t*, and 0 otherwise
- $V_{F_{2},i}(t)$ = 1 if subject *i* is fully vaccinated from BNT162b2 at time *t*, and 0 otherwise
- $V_{B_{11},i}\left( t \right)$ = 1 if subject *i* is boosted from complete mRNA-1273 sequence at time *t*, and 0 otherwise
- $V_{B_{12},i}\left( t \right)$ = 1 if subject *i* is boosted from complete BNT162b2 sequence at time *t*, and 0 otherwise
- $V_{B_{2},i}\left( t \right)$ = 1 if subject *i* has mix-matched booster at time *t*, and 0 otherwise

**Vaccine protection by previous SARS-CoV-2 infection history (across manufacturers)**

$h\left( t | V_{i},P_{i},\boldsymbol{X}_{i} \right)=h_{0}\left( t \right)\times\exp\left\{ \begin{aligned} \alpha\times V_{Pi}\left( t \right)+\beta\times V_{Fi}\left( t \right)+\gamma\times V_{Bi}(t)+\nu\times P_{i}+\delta_{1}\times V_{Pi}\left( t \right)\times P_{i} \\ +\delta_{2}\times V_{Fi}\left( t \right)\times P_{i}+\delta_{3}\times V_{Bi}\left( t \right)\times P\_i+\boldsymbol{\eta}'\boldsymbol{X}_{i} \end{aligned} \right\}$ (Model 1.4)

- Interaction between full vaccination and previous infection: $exp\{\delta_{2}\}$
- Interaction between boosted and previous infection: $exp\{\delta_{3}\}$
