# Supplementary material for: Covid-19 vaccine effectiveness against general SARS-CoV-2 infection from the omicron variant: A retrospective cohort study
Source: PLOS Glob Public Health. 2023 Jan 10;3(1):e0001111. doi: 10.1371/journal.pgph.0001111 (PMC9910751; doi:10.1371/journal.pgph.0001111)
Supplement: S2 Appendix — (DOCX) [file pgph.0001111.s002.docx]

# **S2 Appendix. Statistical models for waning vaccine protection**

**Vaccine protection (across manufacturers):**

$h\left( t | V_{i},P_{i},\boldsymbol{X}_{i} \right)=h_{0}\left( t \right)\times\exp\left\{ \begin{aligned} \alpha_{1}\times V_{Pi}\left( t \right)+\alpha_{2}\times V_{Pi}\left( t \right)\times T_{{V_{P}}_{i}}+\beta_{1}\times V_{Fi}\left( t \right)+\beta_{2}\times V_{Fi}\left( t \right)\times T_{{V_{F}}_{i}}+ \\ \gamma_{1}\times V_{Bi}\left( t \right)+\gamma_{2}\times V_{Bi}\left( t \right)\times T_{V_{Bi}}+\nu_{1}\times P_{i}+\nu_{2}\times P_{i}\times T_{P_{i}}+\boldsymbol{\eta}'\boldsymbol{X}_{i} \end{aligned} \right\}$ (Model 2.1)

- Full vaccination: hazard ratio for change in monthly risk: $\exp\left\{ \beta_{2}\times30 \right\}$
- Booster dose: hazard ratio for change in monthly risk: $\exp\left\{ \gamma_{2}\times30 \right\}$
- $T_{V_{P_{i}}}$ is days between date of partial vaccination and time *t*
- $T_{V_{F_{i}}}$ is days between date of full vaccination and time *t*
- $T_{V_{B_{i}}}$ is days between date of booster and time *t*
- $T_{P_{i}}$ is days between date of previous SARS-CoV-2 infection and time *t*

**Vaccine protection (by manufacturer)**

$h\left( t | \boldsymbol{V}_{i},P_{i},\boldsymbol{X}_{i} \right)=h_{0}\left( t \right)\times\exp\left\{ \begin{aligned} \sum_{k=1:2} \alpha_{1k}\times V_{P_{k},i}\left( t \right)+\sum_{k=1:2} \alpha_{2k}\times V_{P_{k},i}\left( t \right)\times T_{V_{P_{i},k}} \\ +\sum_{l=1:2} \beta_{1l}\times V_{F_{l},i}\left( t \right)+\sum_{l=1:2} \beta_{2l}\times V_{F_{l},i}\left( t \right)\times T_{V_{C_{i},l}}+\sum_{j=1:2} \gamma_{1j}\times V_{B_{j},i}\left( t \right)+ \\ \sum_{j=1:2} \gamma_{2j}\times V_{B_{j},i}\left( t \right)\times T_{V_{B_{i},j}}+\nu_{1}\times P_{i}+\nu_{2}\times P_{i}\times T_{P_{i}}+\boldsymbol{\eta}'\boldsymbol{X}_{i} \end{aligned} \right\}$ (Model 2.2)

- Differences in change in monthly risk between mRNA-1273 and BNT162b2 among fully vaccinated: $H_{0}: \beta_{21}=\beta_{22}$
- Differences in change in monthly risk between mRNA-1273 and BNT162b2 among fully vaccinated: $H_{0}: \gamma_{21}=\gamma_{22}$
- $T_{V_{P_{1},k}}$ is days between date of first dose of mRNA-1273 and time *t*
- $T_{V_{P_{2},k}}$ is days between date of first dose of BNT162b2 and time *t*
- $T_{V_{F_{1},k}}$ is days between date of second dose of mRNA-1273 and time *t*
- $T_{V_{F_{2},k}}$ is days between date of second dose of BNT162b2 and time *t*
- $T_{V_{B_{1},k}}$ is days between date of booster dose of mRNA-1273 and time *t*
- $T_{V_{B_{2},k}}$ is days between date of booster dose of BNT162b2 and time *t*

**Vaccine protection (across manufacturers) with common decline over time**

$h\left( t | V_{i},P_{i},\boldsymbol{X}_{i} \right)=h_{0}\left( t \right)\times\exp\left\{ \alpha\times V_{Bi}\left( t \right)+\beta\times T_{Vi}+\nu_{1}\times P_{i}+\nu_{2}\times P_{i}\times T_{Pi}+\boldsymbol{\eta}'\boldsymbol{X}_{i} \right\}$ (Model 2.3)

- Time-adjusted risk of SARS-CoV-2 infection: $exp\{\alpha\}$
- $T_{Vi}=\left\{ \begin{aligned} days between date of full vaccination and time t, if subject i fully vaccinated \\ days between date of boosted and time t, if subject i \mathrm{boosted} \end{aligned} \right.$

This model is restricted to individuals who were fully vaccinated or boosted at the start of follow-up.

- Due to restricting the sample to fully vaccinated individuals who are not booster eligible during the follow-up period (i.e., received their second dose less than 5.25 months prior to end of follow-up), $V_{Bi}\left( t \right)$ is fixed for each subject *i* in this model (i.e., $V_{Bi}\left( t \right)=V_{Bi})$
