# Supplementary material for: Covid-19 vaccine effectiveness against general SARS-CoV-2 infection from the omicron variant: A retrospective cohort study
Source: PLOS Glob Public Health. 2023 Jan 10;3(1):e0001111. doi: 10.1371/journal.pgph.0001111 (PMC9910751; doi:10.1371/journal.pgph.0001111)
Supplement: S3 Appendix — (DOCX) [file pgph.0001111.s003.docx]

# **S3 Appendix. Study sample description**

Demographic and clinical information for the student and employee populations are presented in Tables S1A and S1B, respectively. The study population includes every main-campus student and employee, with the exception of: individuals not affiliated with the university during the Fall 2021 semester (exclusion rationale provided in Methods > Vaccination Status in the main text), individuals reporting only one dose of Pfizer (BNT162b2) or Moderna (mRNA-1273), or individuals receiving a vaccination dose from a non-mRNA (Pfizer or Moderna) vaccine. Also included in these tables is the population of individuals who were fully vaccinated and booster eligible, but did not report a booster dose. Note that these individuals were removed from the analytic population (exclusion rationale provided in Methods > Vaccination Status in the main text). Tables S1A and S1B compare demographic and clinical characteristics between all vaccination groups. A separate comparison of demographic and clinical characteristics restricted to only vaccination groups included in the analytic population (unvaccinated, booster ineligible fully vaccinated, and boosted groups) are also included.

*Students*

Demographic and clinical variables with substantial clinical differences between groups include gender, residential status, conditions impacting immune response or other pre-existing conditions, use of medications, use of tobacco/nicotine products, previous number of SARS-CoV-2 tests, and previous SARS-CoV-2 infection (Table S1A). The average age of this population was 20.01 years (SD=1.51). The majority of the population was non-Hispanic White (78.9%), female (51.6%), lived in non-residential housing (65.6%), did not report a condition impact immune response (99.2%), any other pre-existing condition (96.3%), or medication use (99.1%), did not use tobacco or nicotine products (94.6%), and did not have a previous SARS-CoV-2 infection (73.0%). The average number of SARS-CoV-2 tests per person since the Fall 2020 semester was 28.44 (SD=13.35). Statistically significant differences were observed among all variables between groups (Table S1A). Most notably, the boosted group had a higher proportion of females, residential students, conditions impacting immune response, other pre-existing conditions, and use of medications relative to other groups. In addition, the boosted group had a higher number of previous SARS-CoV-2 tests (per person) and lower proportion of individuals using tobacco or nicotine products, a lower rate of previous SARS-CoV-2 infection, and a lower rate of SARS-CoV-2 infection during the study follow-up period.

There were some major differences between the study population (Table S1A) and analytic population (Table 1A). In particular, the analytic population had a higher proportion of females, residential students, and previous SARS-CoV-2 infection, and a lower rate of individuals with a condition impacting immune response (these individuals were excluded by design), other pre-existing conditions, medication use (these individuals were excluded by design), and use of tobacco or nicotine products. These differences are due to choosing the boosted group as the common-referent category when matching, which skewed the analytic population characteristics towards that of the boosted group.

*Employees*

Demographic and clinical variables with substantial clinical differences between groups include age, gender, affiliation, conditions impacting immune response or other pre-existing conditions, use of medications, use of tobacco/nicotine products, previous number of SARS-CoV-2 tests, and previous SARS-CoV-2 infection (Table S1B). The average age of this population was 44.19 years (SD=11.69). The majority of the population was non-Hispanic White (78.6%), female (51.9%), staff (66.6%), did not report a condition impact immune response (97.3%), any other pre-existing condition (70.0%), or medication use (97.5%), did not use tobacco or nicotine products (95.4%), and did not have a previous SARS-CoV-2 infection (86.6%). The average number of SARS-CoV-2 tests per person since the Fall 2020 semester was 33.39 (SD=17.48). Statistically significant differences were observed among all variables between groups, with the exception of medication use (Table S1B). Most notably, the booster ineligible fully vaccinated group and boosted group had a higher proportion of females, while the booster eligible fully vaccinated group and boosted group had a higher proportion of faculty. Relative to other groups, the boosted group was older, had a higher proportion of individuals with conditions impacting immune response and other pre-existing conditions. In addition, the boosted group had a higher number of previous SARS-CoV-2 tests (per person) and lower proportion of individuals using tobacco or nicotine products, a lower rate of previous SARS-CoV-2 infection, and a lower rate of SARS-CoV-2 infection during the study follow-up period.

There were some major differences between the study population (Table S1B) and analytic population (Table 1B). In particular, the analytic population had a higher proportion of females, white non-Hispanic population, staff, and previous SARS-CoV-2 infection, and a lower rate of individuals with a condition impacting immune response (these individuals were excluded by design), other pre-existing conditions, medication use (these individuals were excluded by design), and use of tobacco or nicotine products. The analytic population also had a lower number of SARS-CoV-2 tests per person. These differences are due to choosing the booster ineligible fully vaccinated group as the common-referent category when matching, which skewed the analytic population characteristics towards that of the booster ineligible fully vaccinated group.
