# Supplementary material for: Covid-19 vaccine effectiveness against general SARS-CoV-2 infection from the omicron variant: A retrospective cohort study
Source: PLOS Glob Public Health. 2023 Jan 10;3(1):e0001111. doi: 10.1371/journal.pgph.0001111 (PMC9910751; doi:10.1371/journal.pgph.0001111)
Supplement: S1 Table — (DOCX) [file pgph.0001111.s004.docx]

**S1 Table**. Characteristics of student study population (prior to matching). The original student study population consisted of 19,757 students, of which 17,967 are included in Table S1A below. Exclusion criteria includes removal of: 693 students without a university affiliation during the Fall 2021 semester, 136 students reporting only partial vaccination, and 960 students receiving a primary vaccine other than an mRNA (Pfizer or Moderna) vaccine.

| **Characteristic** | **Total**  **N = 17967** | **Unvaccinated**  **N = 7298** | **Fully vaccinated;**  **booster eligible^‣^**  **N = 8765** | **Fully vaccinated;**  **booster ineligible^●^**  **N = 962** | **Boosted**  **N = 942** | **P-value (all groups)^a^** | **P-value (3 groups)^b^** |
| --- | --- | --- | --- | --- | --- | --- | --- |
| **Age: Mean (SD)** | 20.01 (1.51) | 19.98 (1.51) | 20.06 (1.53) | 20.10 (1.43) | 19.72 (1.44) | <0.001 | <0.001 |
| **Race/Ethnicity: N (%)** |  |  |  |  |  |  |  |
| ...White non-Hispanic | 14176 (78.9%) | 5933 (81.3%) | 6713 (76.6%) | 774 (80.5%) | 756 (80.3%) | <0.001 | 0.64 |
| ...Black non-Hispanic | 1151 (6.4%) | 392 (5.4%) | 646 (7.4%) | 70 (7.3%) | 43 (4.6%) | <0.001 | 0.02 |
| ...Any race Hispanic | 1180 (6.6%) | 453 (6.2%) | 598 (6.8%) | 60 (6.2%) | 69 (7.3%) | 0.32 | 0.41 |
| ...All other races non-Hispanic | 1460 (8.1%) | 520 (7.1%) | 808 (9.2%) | 58 (6.0%) | 74 (7.9%) | <0.001 | 0.29 |
| **Gender: N (%)** |  |  |  |  |  |  |  |
| ...Female | 9263 (51.6%) | 3163 (43.3%) | 4899 (55.9%) | 536 (55.7%) | 665 (70.6%) | <0.001 | <0.001 |
| ...Male | 8655 (48.2%) | 4110 (56.3%) | 3845 (43.9%) | 425 (44.2%) | 275 (29.2%) | <0.001 | <0.001 |
| ...Not reported | 49 (0.3%) | 25 (0.3%) | 21 (0.2%) | 1 (0.1%) | 2 (0.2%) | 0.42 | 0.39 |
| **Affiliation: N (%)** |  |  |  |  |  |  |  |
| ...Residential | 6183 (34.4%) | 2450 (33.6%) | 2973 (33.9%) | 278 (28.9%) | 482 (51.2%) | <0.001 | <0.001 |
| ...Non-residential | 11784 (65.6%) | 4848 (66.4%) | 5792 (66.1%) | 684 (71.1%) | 460 (48.8%) | <0.001 | <0.001 |
| **Graduate student: N (%)** | 713 (4.0%) | 167 (2.3%) | 475 (5.4%) | 37 (3.8%) | 34 (3.6%) | <0.001 | 0.002 |
| **Condition impacting immune response: N (%)^$^** | 147 (0.8%) | 46 (0.6%) | 84 (1.0%) | 3 (0.3%) | 14 (1.5%) | 0.004 | 0.004 |
| **Pre-existing condition: N (%)** | 666 (3.7%) | 217 (3.0%) | 377 (4.3%) | 33 (3.4%) | 39 (4.1%) | <0.001 | 0.13 |
| ...High blood pressure | 121 (0.7%) | 49 (0.7%) | 60 (0.7%) | 5 (0.5%) | 7 (0.7%) | 0.94 | 0.82 |
| ...Heart disease | 26 (0.1%) | 10 (0.1%) | 15 (0.2%) | 0 (0.0%) | 1 (0.1%) | 0.58 | 0.51 |
| ...Diabetes | 98 (0.5%) | 35 (0.5%) | 50 (0.6%) | 5 (0.5%) | 8 (0.8%) | 0.51 | 0.33 |
| ...Overweight | 433 (2.4%) | 130 (1.8%) | 254 (2.9%) | 23 (2.4%) | 26 (2.8%) | <0.001 | 0.07 |
| ...Kidney disease | 12 (0.1%) | 4 (0.1%) | 8 (0.1%) | 0 (0.0%) | 0 (0.0%) | 0.53 | 0.59 |
| ...Cough inefficacy | 4 (0.0%) | 0 (0.0%) | 4 (0.0%) | 0 (0.0%) | 0 (0.0%) | 0.24 | NA |
| ...Liver disease | 8 (0.0%) | 2 (0.0%) | 6 (0.1%) | 0 (0.0%) | 0 (0.0%) | 0.48 | 0.77 |
| **Medications: N (%)** | 159 (0.9%) | 47 (0.6%) | 92 (1.0%) | 4 (0.4%) | 16 (1.7%) | <0.001 | <0.001 |
| ...Steroids | 75 (0.4%) | 19 (0.3%) | 42 (0.5%) | 3 (0.3%) | 11 (1.2%) | <0.001 | <0.001 |
| ...Chemotherapy | 4 (0.0%) | 0 (0.0%) | 3 (0.0%) | 0 (0.0%) | 1 (0.1%) | 0.15 | 0.01 |
| ...Immunosuppressants | 91 (0.5%) | 29 (0.4%) | 55 (0.6%) | 1 (0.1%) | 6 (0.6%) | 0.05 | 0.17 |
| **Use of tobacco or nicotine products: N (%)** | 971 (5.4%) | 523 (7.2%) | 373 (4.3%) | 63 (6.5%) | 12 (1.3%) | <0.001 | <0.001 |
| **SARS-CoV-2 Tests Per Person: Mean (SD)** | 28.44 (13.35) | 24.20 (12.10) | 31.08 (13.10) | 30.33 (13.85) | 34.82 (14.96) | <0.001 | <0.001 |
| ...Fall 2020 Semester | 5.33 (3.59) | 4.56 (3.27) | 5.73 (3.65) | 5.20 (3.71) | 7.20 (3.77) | <0.001 | <0.001 |
| ...Spring 2021 Semester | 11.57 (6.66) | 9.91 (6.17) | 12.48 (6.60) | 11.61 (7.31) | 14.81 (7.10) | <0.001 | <0.001 |
| ...Fall 2021 Semester | 14.00 (4.90) | 12.48 (5.33) | 14.91 (4.16) | 14.49 (4.82) | 16.85 (4.28) | <0.001 | <0.001 |
| ...Spring 2022 Semester^¶*^ | 3.17 (1.41) | 2.82 (1.25) | 3.34 (1.44) | 3.14 (1.36) | 4.29 (1.56) | <0.001 | <0.001 |
| **Previous SARS-CoV-2 Infection: N (%)^†^** | 4849 (27.0%) | 2317 (31.7%) | 2048 (23.4%) | 305 (31.7%) | 179 (19.0%) | <0.001 | <0.001 |
| **SARS-CoV-2 Infection During Follow-up: N (%)^#^** | 4929 (27.4%) | 2077 (28.5%) | 2387 (27.2%) | 295 (30.7%) | 170 (18.0%) | <0.001 | <0.001 |

**^‣^** 2^nd^ dose administered > 5.25 months of study end date. These individuals were eligible for a booster dose by end of study date, but did not report a booster dose. Because there was no way of ascertaining whether these individuals never received a booster dose, or received a booster dose but did not report it, these individuals were removed from the analytic population.

**^●^** 2^nd^ dose administered < 5.25 months of study end date (these individuals not eligible for booster dose by end of study date).

^a^ Comparing unvaccinated, booster eligible fully vaccinated, booster ineligible fully vaccinated, and boosted groups

^b^ Comparing unvaccinated, booster ineligible fully vaccinated, and boosted groups

^$^ Self-reported presence of: HIV, Cancer, Lupus, Rheumatoid Arthritis, Solid organ or bone marrow transplant

^†^ Infection occurring prior to follow-up period (1/3/22)

^#^ % is proportion of individuals within each population infected with SARS-CoV-2 during follow-up period

^*^ Variable not included in propensity score model

**Table S1B**. Characteristics of employee study population (prior to matching). The original employee study population consisted of 4,389 employees, of which 3,950 are included in Table S1B below. Exclusion criteria includes removal of: 178 employees without a university affiliation during the Fall 2021 semester, 8 employees reporting only partial vaccination, and 253 employees receiving a primary vaccine other than an mRNA (Pfizer or Moderna) vaccine.

| **Characteristic** | **Total**  **N = 3950** | **Unvaccinated**  **N = 935** | **Fully vaccinated;**  **booster eligible^‣^**  **N = 2303** | **Fully vaccinated;**  **booster ineligible^●^**  **N = 103** | **Boosted**  **N = 609** | **P-value (all groups)^a^** | **P-value (3 groups)^b^** |
| --- | --- | --- | --- | --- | --- | --- | --- |
| **Age: Mean (SD)** | 44.19 (11.69) | 42.67 (12.21) | 44.13 (11.55) | 42.39 (11.47) | 47.03 (10.93) | <0.001 | <0.001 |
| **Race/Ethnicity: N (%)** |  |  |  |  |  |  |  |
| ...White non-Hispanic | 3106 (78.6%) | 731 (78.2%) | 1783 (77.4%) | 84 (81.6%) | 508 (83.4%) | 0.01 | 0.04 |
| ...Black non-Hispanic | 313 (7.9%) | 95 (10.2%) | 181 (7.9%) | 9 (8.7%) | 28 (4.6%) | 0.001 | <0.001 |
| ...Any race Hispanic | 151 (3.8%) | 31 (3.3%) | 97 (4.2%) | 0 (0.0%) | 23 (3.8%) | 0.13 | 0.14 |
| ...All other races non-Hispanic | 380 (9.6%) | 78 (8.3%) | 242 (10.5%) | 10 (9.7%) | 50 (8.2%) | 0.16 | 0.88 |
| **Gender: N (%)** |  |  |  |  |  |  |  |
| ...Female | 2050 (51.9%) | 456 (48.8%) | 1172 (50.9%) | 65 (63.1%) | 357 (58.6%) | <0.001 | <0.001 |
| ...Male | 1891 (47.9%) | 475 (50.8%) | 1128 (49.0%) | 38 (36.9%) | 250 (41.1%) | <0.001 | <0.001 |
| ...Not reported | 9 (0.2%) | 4 (0.4%) | 3 (0.1%) | 0 (0.0%) | 2 (0.3%) | 0.37 | 0.78 |
| **Affiliation: N (%)** |  |  |  |  |  |  |  |
| ...Faculty | 1318 (33.4%) | 147 (15.7%) | 909 (39.5%) | 10 (9.7%) | 252 (41.4%) | <0.001 | <0.001 |
| ...Staff | 2632 (66.6%) | 788 (84.3%) | 1394 (60.5%) | 93 (90.3%) | 357 (58.6%) | <0.001 | <0.001 |
| **Condition impacting immune response: N (%)^$^** | 106 (2.7%) | 24 (2.6%) | 66 (2.9%) | 1 (1.0%) | 15 (2.5%) | 0.66 | 0.61 |
| **Pre-existing condition: N (%)** | 1185 (30.0%) | 262 (28.0%) | 689 (29.9%) | 29 (28.2%) | 205 (33.7%) | 0.12 | 0.06 |
| ...High blood pressure | 645 (16.3%) | 154 (16.5%) | 378 (16.4%) | 14 (13.6%) | 99 (16.3%) | 0.9 | 0.75 |
| ...Heart disease | 52 (1.3%) | 12 (1.3%) | 32 (1.4%) | 1 (1.0%) | 7 (1.1%) | 0.95 | 0.95 |
| ...Diabetes | 178 (4.5%) | 37 (4.0%) | 105 (4.6%) | 3 (2.9%) | 33 (5.4%) | 0.48 | 0.29 |
| ...Overweight | 749 (19.0%) | 155 (16.6%) | 433 (18.8%) | 20 (19.4%) | 141 (23.2%) | 0.01 | 0.006 |
| ...Kidney disease | 12 (0.3%) | 3 (0.3%) | 7 (0.3%) | 0 (0.0%) | 2 (0.3%) | 0.95 | 0.85 |
| ...Cough inefficacy | 6 (0.2%) | 3 (0.3%) | 3 (0.1%) | 0 (0.0%) | 0 (0.0%) | 0.41 | 0.32 |
| ...Liver disease | 14 (0.4%) | 6 (0.6%) | 6 (0.3%) | 1 (1.0%) | 1 (0.2%) | 0.21 | 0.32 |
| **Medications: N (%)** | 98 (2.5%) | 18 (1.9%) | 56 (2.4%) | 4 (3.9%) | 20 (3.3%) | 0.3 | 0.17 |
| ...Steroids | 53 (1.3%) | 11 (1.2%) | 33 (1.4%) | 1 (1.0%) | 8 (1.3%) | 0.93 | 0.95 |
| ...Chemotherapy | 9 (0.2%) | 1 (0.1%) | 6 (0.3%) | 0 (0.0%) | 2 (0.3%) | 0.75 | 0.55 |
| ...Immunosuppressants | 49 (1.2%) | 8 (0.9%) | 27 (1.2%) | 4 (3.9%) | 10 (1.6%) | 0.05 | 0.03 |
| **Use of tobacco or nicotine products: N (%)** | 180 (4.6%) | 85 (9.1%) | 81 (3.5%) | 3 (2.9%) | 11 (1.8%) | <0.001 | <0.001 |
| **SARS-CoV-2 Tests Per Person: Mean (SD)** | 33.39 (17.48) | 30.37 (17.23) | 33.77 (16.78) | 31.91 (15.37) | 36.85 (19.88) | <0.001 | <0.001 |
| ...Fall 2020 Semester | 3.31 (3.91) | 2.96 (3.25) | 3.40 (4.08) | 2.71 (1.78) | 3.53 (4.28) | 0.03 | 0.02 |
| ...Spring 2021 Semester | 13.50 (8.21) | 12.27 (7.65) | 13.69 (8.07) | 12.09 (7.42) | 14.76 (9.34) | <0.001 | <0.001 |
| ...Fall 2021 Semester | 11.20 (5.08) | 9.96 (5.18) | 11.39 (4.78) | 10.49 (4.56) | 12.51 (5.64) | <0.001 | <0.001 |
| ...Spring 2022 Semester^¶*^ | 4.12 (1.66) | 3.75 (1.67) | 4.16 (1.66) | 4.09 (1.58) | 4.53 (1.57) | <0.001 | <0.001 |
| **Previous SARS-CoV-2 Infection: N (%)^†^** | 528 (13.4%) | 219 (23.4%) | 234 (10.2%) | 24 (23.3%) | 51 (8.4%) | <0.001 | <0.001 |
| **SARS-CoV-2 Infection During Follow-up: N (%)^#^** | 696 (17.6%) | 252 (27.0%) | 361 (15.7%) | 26 (25.2%) | 57 (9.4%) | <0.001 | <0.001 |

**^‣^** 2^nd^ dose administered > 5.25 months of study end date. These individuals were eligible for a booster dose by end of study date, but did not report a booster dose. Because there was no way of ascertaining whether these individuals never received a booster dose, or received a booster dose but did not report it, these individuals were removed from the analytic population. **^●^** 2^nd^ dose administered < 5.25 months of study end date

^a^ Comparing unvaccinated, booster eligible fully vaccinated, booster ineligible fully vaccinated, and boosted groups

^b^ Comparing unvaccinated, booster ineligible fully vaccinated, and boosted groups

^$^ Self-reported presence of: HIV, Cancer, Lupus, Rheumatoid Arthritis, Solid organ or bone marrow transplant

^†^ Infection occurring prior to follow-up period (1/3/22)

^#^ % is proportion of individuals within each population infected with SARS-CoV-2 during follow-up period

^*^ Variable not included in propensity score model
