# Supplementary figures and images for: Covid-19 vaccine effectiveness against general SARS-CoV-2 infection from the omicron variant: A retrospective cohort study
Source: PLOS Glob Public Health. 2023 Jan 10;3(1):e0001111. doi: 10.1371/journal.pgph.0001111 (PMC9910751; doi:10.1371/journal.pgph.0001111)

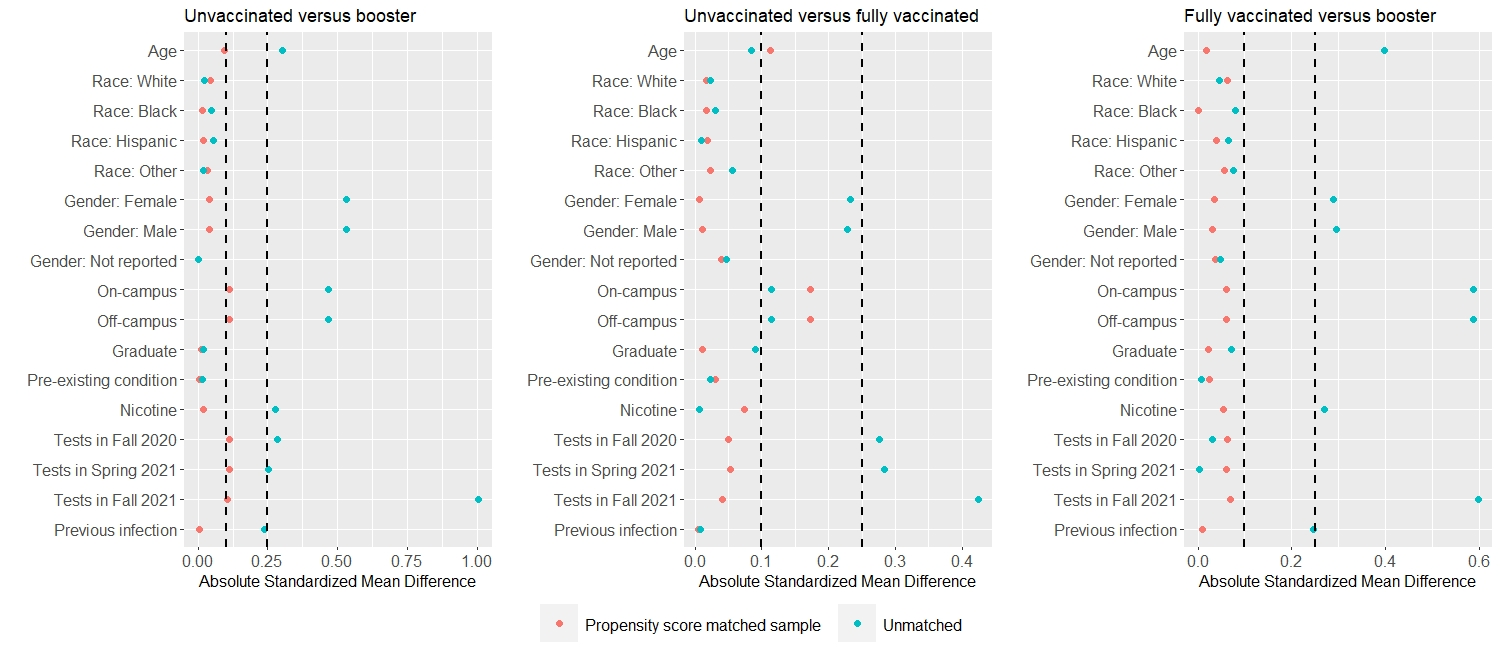

Supplement: S1 Fig — Absolute standardized mean difference between unvaccinated versus booster (left) and unvaccinated versus fully vaccinated (right) for baseline covariates before and after propensity score matching among students. Vertical lines represent the thresholds of 0.1 and 0.25. (TIF) [file pgph.0001111.s006.tif]

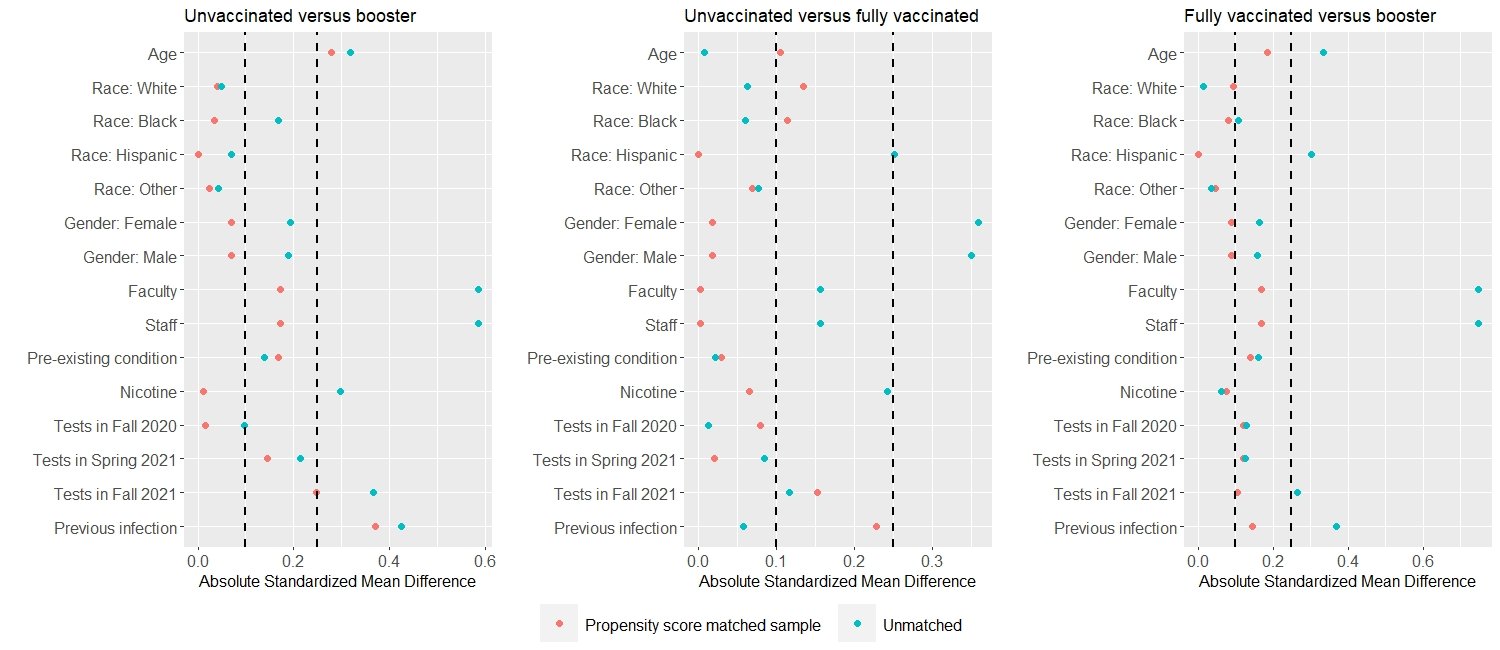

Supplement: S2 Fig — Absolute standardized mean difference between unvaccinated versus booster (left) and unvaccinated versus fully vaccinated (right) for baseline covariates before and after propensity score matching among employees. Vertical lines represent the thresholds of 0.1 and 0.25. (TIF) [file pgph.0001111.s007.tif]

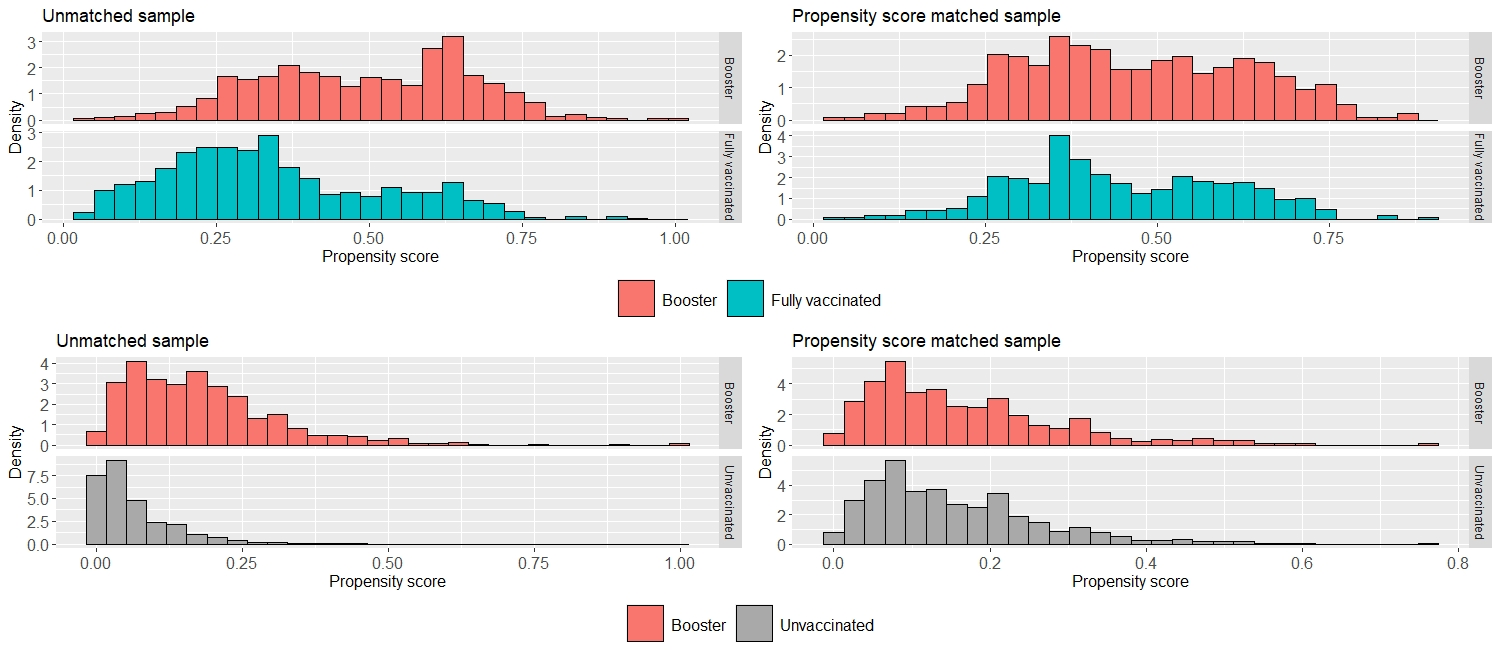

Supplement: S3 Fig — The common reference group (boosted) is represented in red, the unvaccinated group is represented in grey, and the fully vaccinated group is represented in green, respectively. (TIF) [file pgph.0001111.s008.tif]

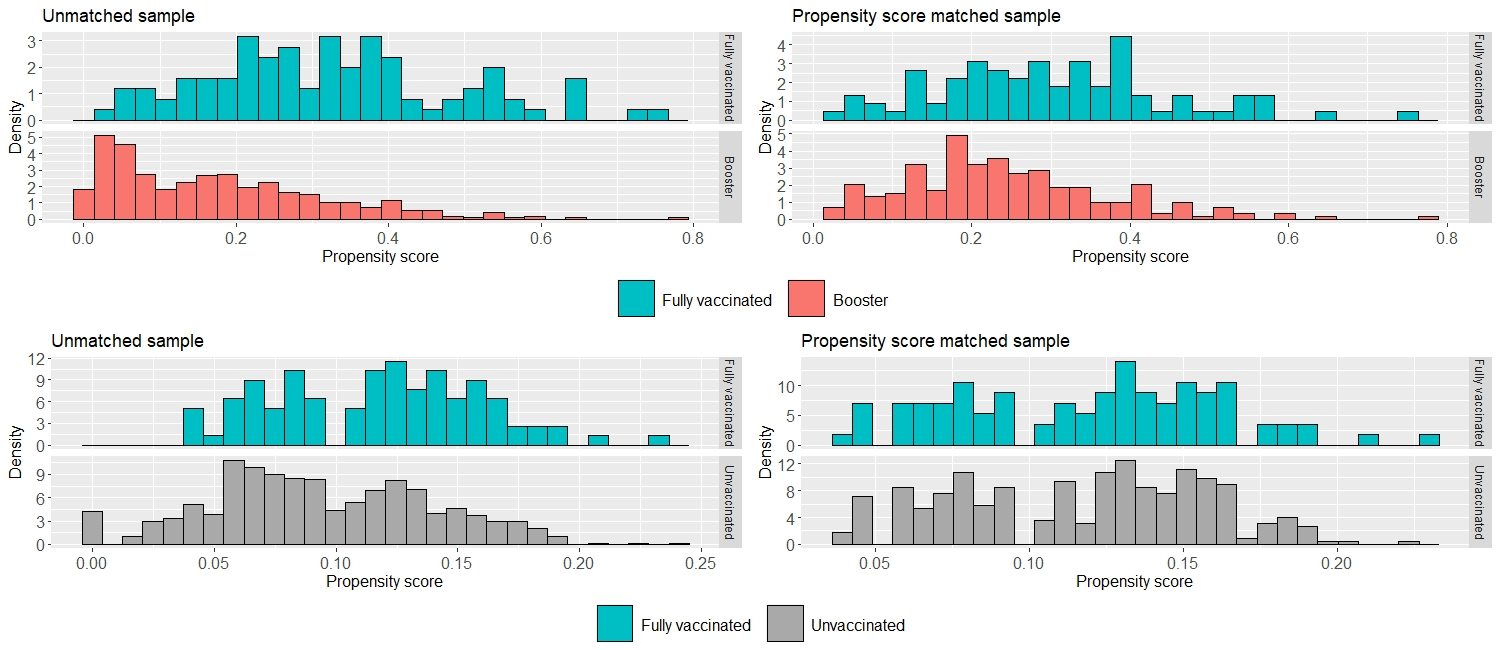

Supplement: S4 Fig — The common reference group (fully vaccinated) is represented in green, the unvaccinated group is represented in grey, and the boosted group is represented in red, respectively. (TIF) [file pgph.0001111.s009.tif]

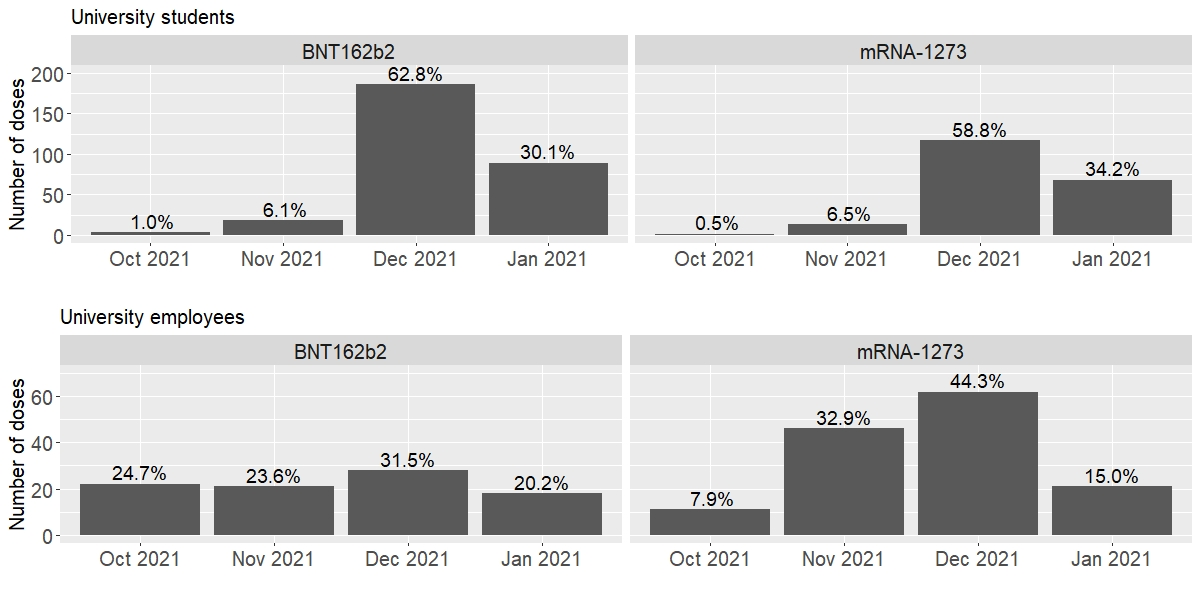

Supplement: S5 Fig — (TIF) [file pgph.0001111.s010.tif]
