# Supplementary material for: Covid-19 vaccine effectiveness against general SARS-CoV-2 infection from the omicron variant: A retrospective cohort study
Source: PLOS Glob Public Health. 2023 Jan 10;3(1):e0001111. doi: 10.1371/journal.pgph.0001111 (PMC9910751; doi:10.1371/journal.pgph.0001111)
Supplement: S1 Checklist — (DOCX) [file pgph.0001111.s011.docx]

**S1 PRISMA Checklist**. STROBE checklist for cohort studies.

|  | Item No | Recommendation | Main Text Page |
| --- | --- | --- | --- |
| **Title and abstract** | 1 | (*a*) Indicate the study’s design with a commonly used term in the title or the abstract | Abstract; page 2 |
|  |  | (*b*) Provide in the abstract an informative and balanced summary of what was done and what was found | Abstract; page 2 |
| Introduction | | |  |
| Background/rationale | 2 | Explain the scientific background and rationale for the investigation being reported | Introduction; page 3 |
| Objectives | 3 | State specific objectives, including any prespecified hypotheses | Introduction; page 3 |
| Methods | | |  |
| Study design | 4 | Present key elements of study design early in the paper | Methods (‘Study Design and Population’); pages 3-4 |
| Setting | 5 | Describe the setting, locations, and relevant dates, including periods of recruitment, exposure, follow-up, and data collection | Methods (‘Study Design and Population’, ‘SARS-CoV-2 Testing’, ‘Vaccination Status’); pages 3-6, Figure 1 |
| Participants | 6 | (*a*) Give the eligibility criteria, and the sources and methods of selection of participants. Describe methods of follow-up | Methods (‘Study Design and Population’, ‘SARS-CoV-2 Testing’, ‘Vaccination Status’); pages 3-6, Figure 1 |
|  |  | (*b*) For matched studies, give matching criteria and number of exposed and unexposed | Methods (‘Propensity Score Matching’); page 6 |
| Variables | 7 | Clearly define all outcomes, exposures, predictors, potential confounders, and effect modifiers. Give diagnostic criteria, if applicable | Methods (‘Statistical Analyses’); pages 6-7 and Table 1A/B |
| Data sources/ measurement | 8* | For each variable of interest, give sources of data and details of methods of assessment (measurement). Describe comparability of assessment methods if there is more than one group | Methods (‘Study Design and Population’, ‘SARS-CoV-2 Testing’, ‘Vaccination Status’); pages 3-6 |
| Bias | 9 | Describe any efforts to address potential sources of bias | Methods (‘Study Design and Population’, ‘Vaccination Status’, ‘Propensity Score Matching’, ‘Statistical Analyses’); pages 3-7 |
| Study size | 10 | Explain how the study size was arrived at | Methods; pages 3-6 and Figure 1 |
| Quantitative variables | 11 | Explain how quantitative variables were handled in the analyses. If applicable, describe which groupings were chosen and why | Methods (‘Propensity Score Matching’, ‘Statistical Analyses’); pages 6-7 and Table 1A/B |
| Statistical methods | 12 | (*a*) Describe all statistical methods, including those used to control for confounding | Methods (‘Propensity Score Matching’, ‘Statistical Analyses’); pages 6-7 and Appendix 1-2 |
|  |  | (*b*) Describe any methods used to examine subgroups and interactions | Methods (‘Statistical Analyses’); pages 6-7 and Appendix 1-2 |
|  |  | (*c*) Explain how missing data were addressed | NA |
|  |  | (*d*) If applicable, explain how loss to follow-up was addressed | Methods (‘Statistical Analyses’). Individuals who did not test positive for SARS-CoV-2 during the follow-up period were censored at their last negative test date; pages 6-7 |
|  |  | (*e*) Describe any sensitivity analyses | NA |
| Results | | |  |
| Participants | 13* | (a) Report numbers of individuals at each stage of study—eg numbers potentially eligible, examined for eligibility, confirmed eligible, included in the study, completing follow-up, and analysed | Results (paragraphs 1-3); pages 7-8, Figure 1, and Table 1A/B |
|  |  | (b) Give reasons for non-participation at each stage |  |
|  |  | (c) Consider use of a flow diagram |  |
| Descriptive data | 14* | (a) Give characteristics of study participants (eg demographic, clinical, social) and information on exposures and potential confounders | Results (paragraphs 1-2); pages 7-8 and Table 1A/B |
|  |  | (b) Indicate number of participants with missing data for each variable of interest | NA |
|  |  | (c) Summarise follow-up time (eg, average and total amount) | Results (‘Vaccine Effectiveness’, paragraphs 1-2); page 9 |
| Outcome data | 15* | Report numbers of outcome events or summary measures over time | Results (paragraphs 1-2); pages 7-8, Table 1A/B, and Table 2 |
| Main results | 16 | (*a*) Give unadjusted estimates and, if applicable, confounder-adjusted estimates and their precision (eg, 95% confidence interval). Make clear which confounders were adjusted for and why they were included | Results (‘Vaccination Effectiveness’, paragraphs 1-3); pages 9-10, Table 2, and Figure 2 |
|  |  | (*b*) Report category boundaries when continuous variables were categorized | NA |
|  |  | (*c*) If relevant, consider translating estimates of relative risk into absolute risk for a meaningful time period | NA |
| Other analyses | 17 | Report other analyses done—eg analyses of subgroups and interactions, and sensitivity analyses | Results (‘Vaccination Effectiveness’, paragraph 4); page 10 |
| Discussion | | |  |
| Key results | 18 | Summarise key results with reference to study objectives | Discussion (paragraphs 1-2); pages 10-11 |
| Limitations | 19 | Discuss limitations of the study, taking into account sources of potential bias or imprecision. Discuss both direction and magnitude of any potential bias | Discussion (paragraphs 5-6); pages 11-12 |
| Interpretation | 20 | Give a cautious overall interpretation of results considering objectives, limitations, multiplicity of analyses, results from similar studies, and other relevant evidence | Discussion (paragraph 7); pages 12-13 |
| Generalisability | 21 | Discuss the generalisability (external validity) of the study results | Discussion (paragraphs 3-6); pages 10-12 |
| Other information | | |  |
| Funding | 22 | Give the source of funding and the role of the funders for the present study and, if applicable, for the original study on which the present article is based | Acknowledgements; page 13 |

*Give information separately for exposed and unexposed groups.
