# Supplementary material for: Covid-19 vaccine effectiveness against general SARS-CoV-2 infection from the omicron variant: A retrospective cohort study
Source: PLOS Glob Public Health. 2023 Jan 10;3(1):e0001111. doi: 10.1371/journal.pgph.0001111 (PMC9910751; doi:10.1371/journal.pgph.0001111)
Supplement: Attachment — Submitted filename: Response to Reviewers.docx [file pgph.0001111.s012.docx]

**Table of Contents**	Pages

Editor and Journal Requirements Responses	2

Reviewer 1 Responses	3-4

Reviewer 2 Responses	5-7

References	8

**Journal Requirements:**

1. Please amend your detailed Financial Disclosure statement. This is published with the article. It must therefore be completed in full sentences and contain the exact wording you wish to be published.
**The authors declare no competing interests.**

b. State what role the funders took in the study. If the funders had no role in your study, please state: “The funders had no role in study design, data collection and analysis, decision to publish, or preparation of the manuscript.”
**We have included the following statement at the end of our Acknowledgements Section: “The funders had no role in study design, data collection and analysis, decision to publish, or preparation of the manuscript.”**

2. Please provide separate figure files in .tif or .eps format.
**These have been provided.**

3. Since your data is not available for proprietary reasons, please explain via email why the data is not available. Please also include the contact information for the third party organization that should be contacted should other researchers want to request access to this data and please include the full citation of where the data can be found. We also request that you verify with us via email that any researcher will be able to obtain the data set in the same manner that the you have obtained it. If you feel you are unwilling or unable to adhere to this policy, please explain your reasons by return email and your exemption request will be escalated to the editor for approval. Your exemption request will be handled independently and will not hold up the peer review process, but will need to be resolved should your manuscript be accepted for publication. One of the Editorial team will be in touch if they require more information.
**We have included the following information in the cover letter: The raw data are protected and are not available due to data privacy laws. Covid-19 data used in this study can be requested at the following link:** [**https://www.clemson.edu/covid-19/testing/research-data.html**](https://www.clemson.edu/covid-19/testing/research-data.html)**.**

Additional Editor Comments (if provided):
Please ensure draft is numbered.
**All documents have been numbered.**

[Note: HTML markup is below. Please do not edit.]

Reviewers' comments:

Reviewer's Responses to Questions

Reviewer #1:

1. I did not find it easy to understand the model chosen to select or exclude participants.

**We have now provided additional detail on the selection/exclusion criteria in the Methods Section. The inclusion criteria consists of all students (18-24 years) and employees (18-65 years) undergoing surveillance testing in January of 2022. Exclusion criteria are described in the Methods Section (*Study Design and Population)* and detailed in Figure 1.**

**There are two additional sets of exclusion criteria that are worth discussing (one of which you mentioned below). First, individuals reporting only 1 dose were excluded from the analysis. This represented only a small fraction of the student (0.67%) and employee (0.24%) populations.**

**The second, more impactful exclusion criteria, pertains to individuals who received 2 doses > five months prior to the end of the study follow-up period BUT did not report a booster dose. The reason these individuals were excluded was to avoid misclassification and reporting bias, arising from the following:**

1. **All university students and employees were provided financial incentives to upload proof of full vaccination, defined as completion of 2-dose mRNA series or 1-dose J&J series, during the Fall 2021 semester**
2. **However, most individuals were not eligible for a booster dose when they provided proof of complete vaccination**
3. **Because there were no incentives to upload the booster dose, there are many students and employees who likely received a booster dose, but never reported it**
4. **Therefore in this study, the group of individuals who were booster eligible (i.e., completed 2-dose series > 5 months ago) consists of those who truly received only 2-doses, but also those who received the booster dose but never reported it.**
5. **Because we have no way of differentiating between these 2 subgroups, we cannot include individuals that were booster eligible but never reported a booster dose**
6. **However, we can include individuals who reported 2-doses < 5 months ago, since these individuals are booster *ineligible* and it is therefore highly unlikely that these individuals received a booster dose**

2. I also fail to understand how in a follow-up period between 3 and 31 January 2022, it has between 27 to 30 tests performed, from which I assume that the follow-up was longer but it is not clear from the text.

**We thank the Reviewer for bringing this to our attention. This number actually refers to the average number of tests *since the Fall 2020 semester* (see Table 1A/1B). The average number of tests during the Spring 2022 follow-up period (Jan 3-Jan 31) was 3.49 for students and 4.07 for employees (Table 1).**

**We believe the confusion arises from the following line in the opening paragraph of the Results Section: “The average number of SARS-CoV-2 tests per person was 30.61 (SD=11.96).” We have therefore clarified that this number refers to the average number of tests since the Fall 2020 semester: “The average number of SARS-CoV-2 tests per person since the Fall 2020 semester was 30.61 (SD=11.96).” This clarification was made for both students and employees.**

3. I also find remarkable the low number of tobacco users, about 2%, which makes me doubt the demographic data (maybe it is a real data, if so, I think it is fantastic).

**It is important to note that the sample descriptives relate to the matched, analytic population, and not the entire student body. Because one of the groups was those voluntarily reporting a booster dose, the analytic sample was more skewed towards a population with health-seeking behavior (note these study designs to balance the sample based on health seeking behavior are widely used in studies evaluating real-world vaccine effectiveness).**^1–3^ **Note that in our previous study evaluating 2-dose vaccine effectiveness against Delta where the majority of the student population was included (no matching was conducted), reported tobacco use was roughly 6%.**^4^

4. Only polymerase chain reaction was used for diagnosis? at the time of the study there were many antigen self-tests available which may have also altered the possible interpretation of results. This is not mentioned on the text

**Our students and employees were required to test weekly at either our university facility or upload a PCR test result. While it is true that at-home antigen tests started to become available during this time period, we did not accept antigen tests. Therefore, while it is possible that individuals may have taken this test, they would have still been required to test at the university facility or upload an approved PCR test. We now mention this in the Methods Section (*SARS-CoV-2 Testing*)**

5. Another point that seems to me of utmost importance and that is mentioned in the discussion is the reporting error that may exist in those who did receive a booster, since there is no economic incentive, so many participants could have had a booster and not be reported, which alters the interpretation of the data.

**The Reviewer is absolutely correct that in the university population, there are many individuals who received a booster but did not report it. For this reason, all individuals who were booster eligible, but did not report it, were excluded from the analysis. A detailed description and rationale is provided in the response to comment #1.**

Reviewer #2:

**We thank the Reviewer for their thoughtful comments and helpful suggestions, which our team believes have helped strengthen both the study and the manuscript. We have responded to all recommendations and suggestions below. Note that in a few instances where we did not proceed with implementing a suggestion, we have provided a rationale.**

Abstract
1. SARS-CoV-2? Write in full then after abbreviate. **Corrected**
2. COVID-19? Write in full then after abbreviate. **Corrected**
3. “Unvaccinated” should be written “unvaccinated” **Corrected**
4. within “the” previous 5 months **Corrected**
5. Intervention should be well described, what were the BNT162b2 or mRNA-1273 vaccine doses? **It is now specified that these are Covid-19 vaccine doses.**
6. Results: How many university students and employees did you include in your results? sex and age distribution should be included. **The number of university/students in both the population and analytic sample are described in Abstract -> Participants. We have also added age, sex, and race distributions separately for student and employee populations.**
7. The result is poorly reported. Please include the BNT162b2 and mRNA-1273 vaccine effectiveness in the result. **Because differences in vaccine manufacturer were not statistically significant, we have refrained from reporting these in the main results. Rather, we report specific vaccine effectiveness estimates of any mRNA vaccination. Estimates by manufacturer can be found in Table 2.**
8. Conclusion: your conclusion should be based on the study population. What are your recommendation based on your results? **We have clarified that the conclusion is that booster doses provide a substantial increase in protection relative to the 2-dose vaccination series for general, otherwise healthy adults 18-65 years of age. However, our team believes that specific recommendations should come from state, national, and international health agencies. Here, we provide quality data and analysis that health agencies can use to inform their decision.**

Introduction
Syndrom should be written “syndrome” **Corrected**
Omicron has caused “a” record number of daily…. **Corrected**
The epidemiology of COVID-19 in the US is poorly described. **Disease epidemiology and etiology are now discussed in the opening paragraph.**
Please briefly review the vaccine effectiveness of Wuhan, beta, and delta variants in the US. Compared this with recent studies conducted about Omicron variant. **Vaccine effectiveness against previous variants has been added in the opening paragraph. Furthermore, we discuss recent studies conducted about the Omicron variant in the Introduction and Discussion Sections.**
Please state clearly the objective of this study and how this study can be considered as particular compared to other studies conducted in the US about Omicron variant. **This is stated at the end of the Introduction. The purpose of this study is to assess vaccine effectiveness against general infection from the Omicron Variant. As stated in the opening paragraph of the Introduction, studies of vaccine protection against *general* infection from omicron are lacking.**
we assess “the” effectiveness of the 2-dose and 3-dose messenger RNA (mRNA) vaccination
“Adminstration” should be written “administration” **We have corrected the typo (but have left the word capitalized for consistency with FDA title).**

Methods
1. The method should include a list of all sociodemographic and covariate variables used in the results.
**All sociodemographic variables and covariates are described in the Methods Section (*Statistical Analysis)* and listed in Tables 1A/1B.**

2. “Time-varying Cox proportional hazard models were used to estimate the relative risk (RR)”, this use to estimate the hazard risk rate.
**The Reviewer is correct that the regression coefficients in the Cox model correspond to the hazard rate. We made the appropriate adjustments throughout the manuscript.**

3. I would like you to compute non adjusted and adjusted RR to estimate the vaccine effectiveness.
**We have included the unadjusted HR in Table 2. We note that the estimates are nearly identical, which is to be expected due to our matched study design.**

4. As the p-value and the 95%CI are included in the results, this should be well stated in the statistical analysis. **This has now been added to the Methods (*Statistical Analysis)* Section**

5. Kind state in the methods that 1-aRR was used to compute the vaccine effectiveness.
**This is now stated in the Methods Section (*Statistical Analysis*), as 1-aHR.**

6. Be consistent between COVID-19 and Covid-19 throughout the study. **We found one instance of COVID-19. The manuscript is now consistent with Covid-19 throughout.**

7. I do not see anything about data collection and management. Please specify.
**We have added a subsection for Data Collection and Management in the Methods Section.**

Results
1. As stated in the abstract, the socio-demographic characteristics should be included in your results. **Sociodemographic characteristics are stated in the first two paragraphs of the Results Section and in Tables 1A and 1B.**

2. Age should be classified because the Anova of unvaccinated, fully vaccinated, and booster was statistically significant. It will also be interesting to see how vaccine effectiveness varies by age distribution in this population.
**While there was a statistically significant difference in mean age between the employee populations (mean age across groups: 42, 43, and 45), this difference is not clinically significant. Furthermore, the average age for each subgroup falls in the 40-49 age range used by the US Centers for Disease Control and Prevention (CDC) to evaluate Covid-19 risk across age groups.^11^ While we agree that vaccine effectiveness by age group would certainly be interesting, our sample size is not sufficient for such calculations. That said, age is controlled for as a covariate in all analyses. We note that the difference in unadjusted and adjusted estimates of vaccine protection were minimal due to the (relatively) successful balancing achieved through weighting.**

2. As stated in the methodology, adjusted RR should be used.
**Adjusted hazard ratio (aHR) is now used throughout.**

Discussion
1. The discussion should be strengthened. How do you compare this study results to other studies related to Omicron vaccine effectiveness? **We compare other study results in the second paragraph of the Discussion Section: Compared to our study, recent studies have found higher 2-dose and 3-dose mRNA vaccine effectiveness against symptomatic and severe SARS-CoV-2 infection from omicron.^8–10^ This is to be expected, since this study evaluates protection against any SARS-CoV-2 infection from omicron (asymptomatic, pre-symptomatic, and symptomatic) as opposed to severe infection.**
2. Authors just state the study limitations. Where are the study strengths? **The study strengths are listed in the paragraph prior.**
3. Clearly state how misclassification of vaccination status may bias estimates of vaccine effectiveness. **We have now clarified that misclassification will attenuate vaccine effectiveness towards the null.**

Conclusion
1. The study recommendation for this specific population should be clearly stated.
**Our study conclusions imply that individuals who receive a booster dose would obtain an increase in protection against general SARS-CoV-2 infection from omicron. However, it is not the objective of this paper to make a specific recommendations on which populations should receive a booster and when. These recommendations should come from state, national, and international health agencies. Here, we provide quality data and analysis that health agencies can use to inform their decision.**

References
1. Pubmed should be used for referencing. Many of the references are incorrect.
**We have found several instances of incomplete or outdated references and have made appropriate corrections.**

**References**

1.	Dagan N, Barda N, Kepten E, et al. BNT162b2 mRNA Covid-19 Vaccine in a Nationwide Mass Vaccination Setting. *New England Journal of Medicine*. 2021;0(0):null. doi:10.1056/NEJMoa2101765

2.	Thompson MG, Stenehjem E, Grannis S, et al. Effectiveness of Covid-19 Vaccines in Ambulatory and Inpatient Care Settings. *New England Journal of Medicine*. 2021;0(0):null. doi:10.1056/NEJMoa2110362

3.	Dean NE, Hogan JW, Schnitzer ME. Covid-19 Vaccine Effectiveness and the Test-Negative Design. *New England Journal of Medicine*. 2021;0(0):null. doi:10.1056/NEJMe2113151

4.	Rennert L, Ma Z, McMahan CS, Dean D. Effectiveness and protection duration of Covid-19 vaccines and previous infection against any SARS-CoV-2 infection in young adults. *Nat Commun*. 2022;13(1):3946. doi:10.1038/s41467-022-31469-z

5.	Bruxvoort KJ, Sy LS, Qian L, et al. Effectiveness of mRNA-1273 against delta, mu, and other emerging variants of SARS-CoV-2: test negative case-control study. *BMJ*. 2021;375:e068848. doi:10.1136/bmj-2021-068848

6.	Singer SR, Angulo FJ, Swerdlow DL, et al. Effectiveness of BNT162b2 mRNA COVID-19 vaccine against SARS-CoV-2 variant Beta (B.1.351) among persons identified through contact tracing in Israel: A prospective cohort study. *eClinicalMedicine*. 2021;42. doi:10.1016/j.eclinm.2021.101190

7.	Chemaitelly H, Tang P, Hasan MR, et al. Waning of BNT162b2 Vaccine Protection against SARS-CoV-2 Infection in Qatar. *New England Journal of Medicine*. 2021;0(0):null. doi:10.1056/NEJMoa2114114

8.	Collie S, Champion J, Moultrie H, Bekker LG, Gray G. Effectiveness of BNT162b2 Vaccine against Omicron Variant in South Africa. *New England Journal of Medicine*. 2021;0(0):null. doi:10.1056/NEJMc2119270

9.	Abu-Raddad LJ, Chemaitelly H, Ayoub HH, et al. Effect of mRNA Vaccine Boosters against SARS-CoV-2 Omicron Infection in Qatar. *New England Journal of Medicine*. 2022;0(0):null. doi:10.1056/NEJMoa2200797

10.	Accorsi EK, Britton A, Fleming-Dutra KE, et al. Association Between 3 Doses of mRNA COVID-19 Vaccine and Symptomatic Infection Caused by the SARS-CoV-2 Omicron and Delta Variants. *JAMA*. Published online January 21, 2022. doi:10.1001/jama.2022.0470

11.	CDC. Cases, Data, and Surveillance. Centers for Disease Control and Prevention. Published February 11, 2020. Accessed August 12, 2022. https://www.cdc.gov/coronavirus/2019-ncov/covid-data/investigations-discovery/hospitalization-death-by-age.html
