# Supplementary material for: Covid-19 vaccine effectiveness against general SARS-CoV-2 infection from the omicron variant: A retrospective cohort study
Source: PLOS Glob Public Health. 2023 Jan 10;3(1):e0001111. doi: 10.1371/journal.pgph.0001111 (PMC9910751; doi:10.1371/journal.pgph.0001111)
Supplement: Attachment — Submitted filename: Response to Reviewers.docx [file pgph.0001111.s013.docx]

Please include the following items when submitting your revised manuscript:

- A rebuttal letter that responds to each point raised by the editor and reviewer(s). You should upload this letter as a separate file labeled 'Response to Reviewers'.
  This has been uploaded.
- A marked-up copy of your manuscript that highlights changes made to the original version. You should upload this as a separate file labeled 'Revised Manuscript with Track Changes'.
  This has been included.
- An unmarked version of your revised paper without tracked changes. You should upload this as a separate file labeled 'Manuscript'.
  This has been included.

Journal Requirements:

1. Please review your reference list to ensure that it is complete and correct. If you have cited papers that have been retracted, please include the rationale for doing so in the manuscript text, or remove these references and replace them with relevant current references. Any changes to the reference list should be mentioned in the rebuttal letter that accompanies your revised manuscript. If you need to cite a retracted article, indicate the article’s retracted status in the References list and also include a citation and full reference for the retraction notice.


We have gone through all 48 references of this paper, and all are correct. In one instance, we have updated the reference since the article went from in-press/online only to journal publication (reference 15). In another instance, we updated the “Article Access Date” in order to get the latest Covid-19 case numbers worldwide (reference 2).

**Reviewer #1:**

1. Don’t use Spring or fall "...in a large public university student and employee population undergoing mandatory weekly testing during the Spring 2022 semester" do not use spring or autumn as a measure of a time period. It is only used in the northern hemisphere and is confusing for those who do not live in that hemisphere. Use the months of the year, which are the same for everyone.

We thank the Reviewer for raising this excellent point. While Spring/Fall semesters are indeed the widely used definitions in the northern hemisphere, they also serve as convenient notation to differentiate between time periods. Differentiation between time periods, even within the same year, is necessary due to major changes in the pandemic (e.g., testing strategies, vaccinations, variants, etc).

That said, we agree with the Reviewer that such labels may be confusing to those who are not familiar with the terms. While at first glance labeling the semesters by months is reasonable, it does create confusion throughout the manuscript because the follow-up period, testing periods, vaccination periods, etc. do not necessarily span the entire time periods.

We have therefore proceeded to make the following changes. In the introduction, we have replaced Spring Semester ’22 with the explicit follow-up period (January 2022). In the methods section, where spring/fall semester labels are necessary to distinguish between different protocols and procedures, we have explicitly defined the spring and fall semester labels and provided approximate date ranges. This is incorporated into the last sentence of the first paragraph of the Methods/Vaccination Status Subsection, where we first introduce fall/spring semester.

2. It is possible to understand, but difficult to do so, the criteria of inclusion and exclusion of the participants, I suggest to improve the wording.

We thank the reviewer for pointing this out. We have modified the verbiage in the Vaccination Status Subsection to make this clearer. We illustrate the inclusion/exclusion criteria in Figure 1. We have also added additional supplementary tables (Appendix 4, Tables S1A/B) that provide information on the major excluded group from this analysis (fully vaccinated booster eligible individuals).

3. How can it be explained that unvaccinated individuals had fewer infections than those vaccinated with two doses? "Compared to fully vaccinated (34.5%) and unvaccinated individuals (31.3%)".

There are several important points here. First, this study, and others, have shown limited effectiveness of 2-dose vaccination series. Second, these are unadjusted estimates. Our previous work has shown that those who were unvaccinated were more likely to have an infection. Therefore, the unvaccinated group contains a disproportionate number of individuals with a previous infection, which is known to offer some protection against future infection. This is the rationale for implementing the adjusted statistical models (Table 2).

4. It would be interesting to know which omicron subvariant was circulating at that date in that place.

During this time, the estimated prevalence of B.1.1.529 (also referred to as BA.1) was over 99%. We have now clarified this in abstract and in the last sentence of Methods/SARS-CoV-2 testing: It is estimated that 99.2% of all SARS-CoV-2 infections in South Carolina were caused by the original omicron variant (BA.1) in January of 2022

**Reviewer #2:** The authors have worked hard to improve the manuscript. The majority of my previous comments have been well addressed. However, the discussion remains poorly described. How do you discuss the statistically significant P-values in table 1A? 1B? and the comparison between students and employees in this study, which was conducted in a university setting? Please make this clear in the discussion.

We thank the Reviewer for the positive feedback and suggestions. The results presented in Tables 1A and 1B are from the post-matched analytic sample. Such matching was necessary due to differences in health-seeking behavior^1^ between unvaccinated, fully vaccinated, and boosted individuals. Without balancing on baseline characteristics, there is a risk of confounding of the vaccination effect on protection from SARS-CoV-2 infection. The p-values are not discussed because these compare characteristics across vaccination groups in a post-matching population.

However, the Reviewer does raise an important point that an overview of the study population should be given. Therefore, following reporting guidelines for propensity score analysis,^2^ we have added a section in the Supplementary materials (Appendix 3) where we both describe the study populations, differences in characteristics between the vaccination group in the study population, and a comparison of the study population to the analytic population. We provide detailed Tables for the student and employee populations in Tables S1A and S1B of the Supplementary Material.

Importantly, we add a paragraph to the Discussion Section (final paragraph of limitations) where we emphasize that not only this is a university population, but the analytic sample differs in some ways from the underlying population. Specifically, that due to the matching protocol, the resulting analytic population tended to be slightly healthier than the underlying study population. We also discuss the uniqueness of university student populations and how they may differ from other young adult populations, and caution that estimates of vaccine effectiveness from this study may not directly translate to other populations. We also discuss and compare the most critical difference between students and employees (age) as it relates to the question of interest: vaccine effectiveness. This can be found in the 3^rd^ paragraph of the discussion section. Specifically, we discuss why we may see worse vaccine protection in the student population, even though they are substantially younger than the employee population.

**References**

1.	Dean NE, Hogan JW, Schnitzer ME. Covid-19 Vaccine Effectiveness and the Test-Negative Design. *New England Journal of Medicine*. 2021;385:1431-1433. doi:10.1056/NEJMe2113151

2.	Yao XI, Wang X, Speicher PJ, et al. Reporting and Guidelines in Propensity Score Analysis: A Systematic Review of Cancer and Cancer Surgical Studies. *J Natl Cancer Inst*. 2017;109(8):djw323. doi:10.1093/jnci/djw323
